# Tricuspid Valve Prolapse: An Uncommon Pathology Revealed by TEE

**DOI:** 10.14797/mdcvj.1095

**Published:** 2022-06-03

**Authors:** David Lewandowski, Faisal Nabi

**Affiliations:** 1 Houston Methodist DeBakey Heart & Vascular Center, Houston, Texas, US

**Keywords:** tricuspid valve prolapse, myxomatous degeneration, tricuspid regurgitation, mitral valve prolapse

## Abstract

A 73-year-old male with a history of dilated cardiomyopathy and paroxysmal atrial fibrillation underwent transthoracic echocardiography (TTE) to evaluate for endocarditis due to fever and gram-positive cocci in chains on blood cultures. TTE revealed a 3 × 8 mm mass on the ventricular aspect of the tricuspid valve (***Figure 1A***). Subsequent transesophageal echocardiography (TEE) showed that the mass in question was actually myxomatous degeneration of the tricuspid valve (TV) and redundant chordae with significant valve prolapse. ***Figure 1B*** shows the prolapsing TV leaflets at the same level as the mitral valve. ***Figure 1C*** and ***1D*** show the valve at the level of the annulus in early systole and then prolapsing 8 mm in mid-late systole, respectively.

Tricuspid valve prolapse (TVP) is uncommon, and one study of 118,000 patients reported an incidence of 0.3%.^1^ Since diagnostic parameters are not clearly defined, diagnosis is often determined subjectively. One objective criteria, > 2 mm atrial displacement of the TV leaflets in the TEE parasternal short-axis view, is noted to have high diagnostic accuracy. TVP is commonly associated with mitral valve prolapse. Patients with TVP have more severe tricuspid regurgitation and right-sided chamber enlargement compared to patients with no TVP. Due to the lack of significant tricuspid regurgitation in this case, the patient was reassured, and no further intervention was recommended.

**Figure 1:**
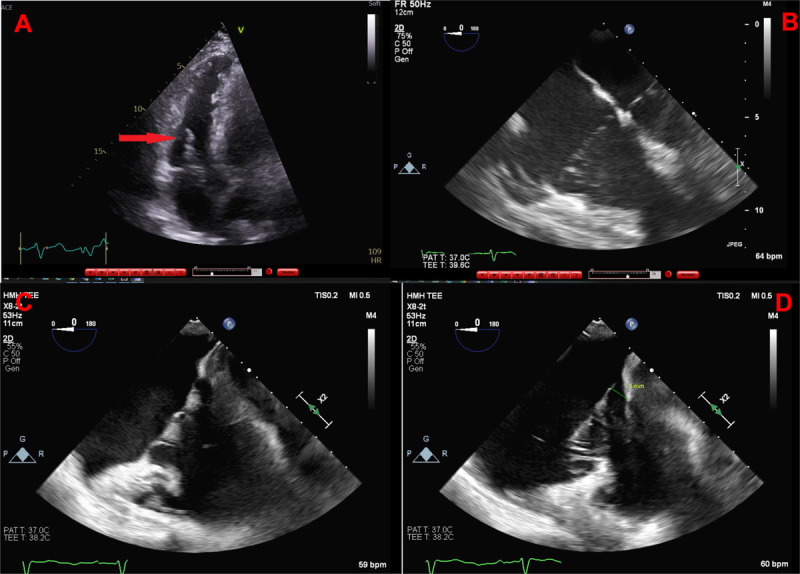
**(A)** Transthoracic echocardiography image demonstrates an apparent echo density on the tricuspid valve. **(B)** Transesophageal echocardiography (TEE) image demonstrates tricuspid prolapse with the mitral and tricuspid valves at equal level in systole. **(C)** TEE image shows the tricuspid leaflets in early systole at the level of the annulus. **(D)** TEE image shows the tricuspid leaflets in late systole prolapsing 8 mm into the right atrium.
